# A map of high-altitude wetlands in the world’s major mountain regions

**DOI:** 10.1038/s41597-026-07020-w

**Published:** 2026-03-11

**Authors:** Rike Becker, Jan Kropáček, Anthony C. Ross, Tom Gribbin, Fabian Drenkhan, Lilia Hernandez Sotelo, Marc Martinez Mendoza, Bethan Davies, Jeremy Ely, Wouter Buytaert

**Affiliations:** 1 Department of Civil and Environmental Engineering, Imperial College London, London, UK; 2 Department of Physical Geography and Geoecology, Faculty of Science, Charles University, Prague, Czechia; 3 British Geological Survey, Environmental Science Centre, Keyworth, UK; 4 School of Geography, Earth and Environmental Sciences, University of Birmingham, Edgbaston, UK; 5 Geography and Environment, Department of Humanities, Pontificia Universidad Católica del Perú, Lima, Peru; 6 Grupo de Glaciología y Ecohidrología de Montañas Andinas (GEMS), Institute for Nature, Earth and Energy (INTE), Pontificia Universidad Católica del Perú, Lima, Peru; 7 City Planning Labs, The World Bank Group, London, UK; 8 Port de Barcelona, Barcelona, Catalunya Spain; 9 School of Geography, Politics and Sociology, Newcastle University, Newcastle-upon-Thyne, UK; 10 School of Geography and Planning, The University of Sheffield, Sheffield, UK

**Keywords:** Hydrology, Environmental sciences

## Abstract

We present a first global high-resolution map (30 m x 30 m) of high-altitudinal wetlands in the world’s major mountain regions, i.e. the Andes, Rocky Mountains, Alps and High Mountain Asia. To map these wetlands, we employed a supervised classification approach using a random forest machine learning model and a selected set of predictors including vegetation, topographic, and surface moisture features. The predictors were derived from freely available radar and optical satellite imagery (Sentinel-1 and Sentinel-2), SRTM elevation data, and the global ecoregion map RESOLVE. We identify a total area of >30,500 km^2^ of high-mountain wetlands. With this map we aim to enhance the understanding of wetland distribution in remote and often inaccessible mountain regions and enable a more reliable understanding of their role in the ecosystem functioning and water cycles of high mountain areas.

## Background & Summary

High mountain wetlands provide a wide range of ecological, environmental, cultural and socio-economic services^1–4^. They serve as important carbon stores^5^, and provide areas for livestock farming and cultural activities for local communities^2^. In addition, they are key to securing safe freshwater supply both for streamflow regulation and water quality, in high mountain and downstream areas. This is particularly relevant where water storage capacities of glaciers and snow fields are shrinking and water resources are increasingly at risk^3,4,6,7^.

We define high mountain wetlands as areas of high moisture accumulation, located above the tree line and below the permanent snow line^3^. These wetlands typically form in flat or gently sloping fluvial or glaciated valley bottoms, where water retention creates areas of high moisture saturation on the land surface^7^. This enables the development of diverse aquatic ecosystems in harsh, rocky, and steep environments. Plant communities in these wetlands are uniquely adapted to extreme environmental conditions and are typically dominated by sedges, rushes, mosses, and cushion plants, which enable carbon sequestration and create hotspots of biodiversity^8–10^. The combination of hydrological and biological features makes them one of the most productive ecosystems in high mountain areas^10^. Depending on their proximity to glaciers and snow fields, wetlands may be sustained by a combination of precipitation, groundwater flow, snow melt, and glacier runoff. Acting as natural buffers, they store water during wet seasons and gradually release it during dry periods, providing critical water resources to social-ecological systems^4,6^.

Despite the importance of high mountain wetlands for ecohydrological processes, detailed spatial information barely exists. Global land cover maps effectively classify swamps, marshes, coastal and lowland wetlands but they lack sufficient detail to accurately capture high mountain wetlands^11,12^. Similarly, high-resolution land cover maps^13^ fall short in this regard. Even the Global Wetlands Map^14^ primarily focuses on lowland wetlands, marshes, swamps, and floodplains, underestimating the presence of wetlands in high mountain regions. To our knowledge, no attempt has been made yet to create a global, high-resolution, and seamless map of mountain wetlands.

Many local wetland classification studies show promising results in identifying mountain wetland extent and its temporal dynamics based on remote sensing imagery^7,15,16^. These studies use spectral information acquired from radar or optical satellite images, together with topographic, hydrological and ecological information to derive surface characteristics specific to mountain wetlands. Yet, these local studies are often of limited geographic extent, and their classification approaches are focussed on specific local wetland characteristics (e.g. detecting green areas vs. bare surroundings^7^). This makes the approach unsuitable for a wetland identification and mapping across global mountain regions with diverse wetland characteristics (e.g. for regions with competing greenness from surrounding vegetation). With the presented mapping approach, we aim at bridging the gap between global maps which offer global land–use and–land cover data but remain limited to map high mountain wetlands, and local wetland maps which are accurate and of high resolution but often specific to their local environment.

We present a high-resolution wetland map (30 m × 30 m) covering the Andes, Rocky Mountains, Alps, and High Mountain Asia. A unified supervised classification approach is applied across all four selected mountain regions. This ensures that our method is consistent across regions and captures the diverse characteristics of wetland ecosystems in these distinct mountain landscapes. Our goal is to produce a wetland map that supports high mountain water resources and ecosystem research, particularly studies on the impacts of climate change on ecosystem functioning, natural capital, and ecosystem services. Our global map reveals that the total area of high-altitude wetlands in the four major mountain regions may exceed 30,500 km^2^. This extent represents a significant carbon reservoir and underlines the importance of these ecosystems for the global environment. The total area also highlights their role as critical water sources in regions increasingly affected by diminishing water supplies due to glacier and snow melt reduction.

## Methods

The wetland map is computed in Google Earth Engine^17^ using a supervised classification approach that integrates multiple data sources: i. Spectral information from Sentinel-1^18,19^ and Sentinel-2^20,21^ satellite imagery; ii. Topographic information derived from the 30 m NASA SRTM Digital Elevation Model (DEM)^22,23^; and iii. global ecoregion data from the RESOLVE data set^24,25^.

**Fig. 1:**
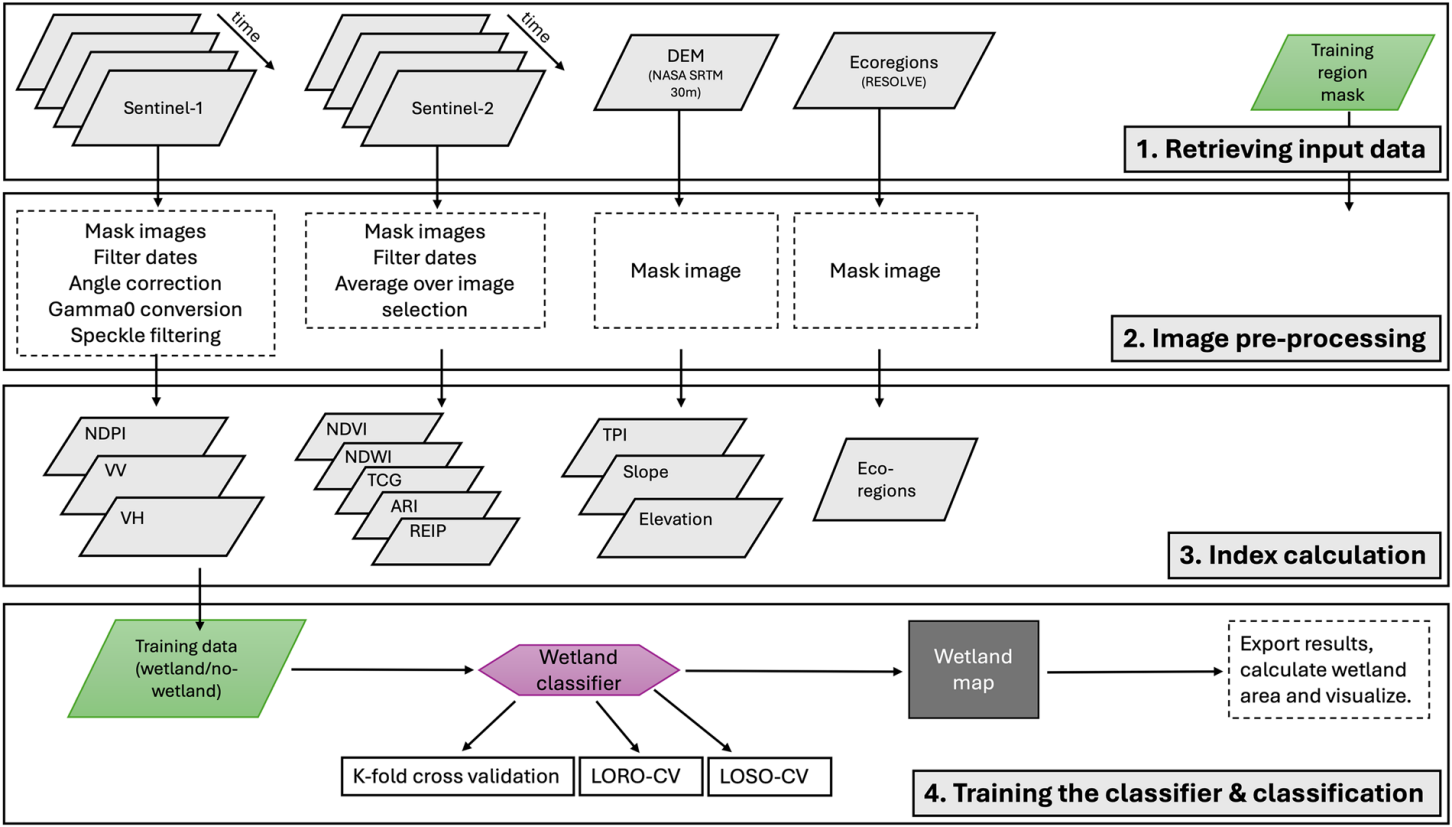
Classification workflow.

We trained a random forest machine learning model on a stratified and random sample of training points. The training data were obtained across high mountain regions in the Andes, Rocky Mountains, Alps and High Mountain Asia, and encompass wetland and non-wetland areas. The model is validated in a k-fold cross validation and its physical plausibility is examined using Shapley values. The classification workflow is detailed in Fig. 1 and further outlined in the subsequent sections.

### Training and validation data

**Fig. 2:**
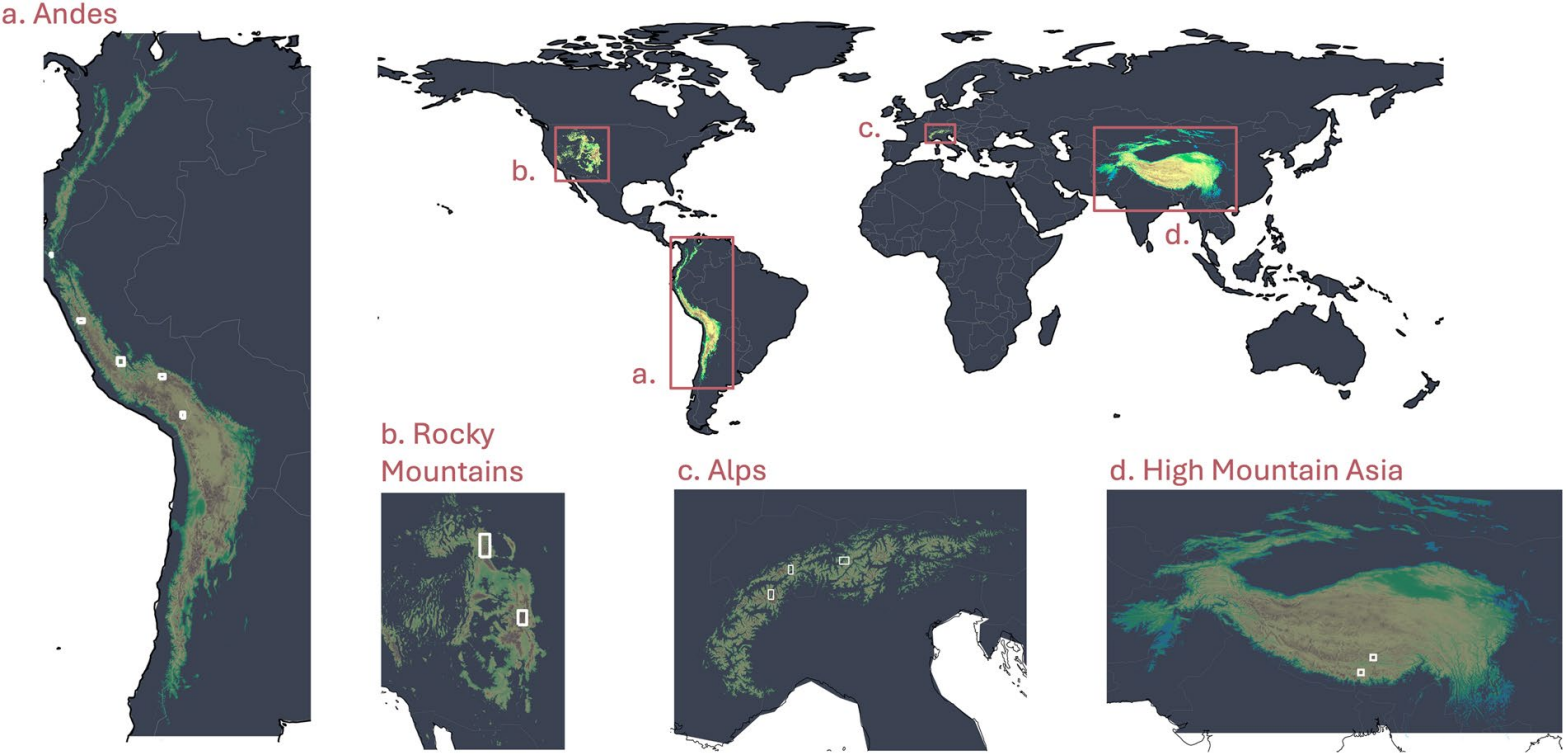
Maps of the 12 test sites (white boxes) across the four selected mountain regions (red boxes).

We chose 12 test sites from which we sampled binary training and validation data of wetlands and non-wetlands (Table 1, Fig. 2). Five test sites are located in the Andes, two in the Rocky Mountains, three in the Alps, and two in High Mountain Asia. Criteria for the selection of test sites were: i. the availability of existing wetland maps and/or local knowledge of wetland distribution; and ii. their spread over several ecoregions^25^. The latter criterion is important to ensure that the sample points used to train the classification algorithm cover a broad spectrum of ecoregion-dependent wetland features. This allows for the training of a model applicable across global mountain regions. The areal extent and location of regional training sites is displayed in Fig. 2 and further quantified in table S.1., which gives additional information on the wetland coverage in each of the 12 sites (see Supplementary Information).

**Table 1:** Data sets for training and validation data for selected ecoregions^25^ and their abbreviation used hereafter.

Mountain region	Ecoregion & characteristics	Source of training data (year of data set)
Andes	Cordillera Central páramo (‘andes_paramos’). Cold and wet climate. Shrubby alpine grassland. Ecoregion ID = 590.	INAIGEM, Peru^26^ (2023)
	Central Andean wet Puna. High mountain plateau (‘andes_wet_puna’). High diurnal temperature variation. Rainy season in summer, dry season in winter. Ecoregion ID = 589.	
	Central Andean Puna. Montane grassland and shrubs (‘andes_puna’). Drier compared to wet Puna. Ecoregion ID = 588.	
	Peruvian Yungas ('andes_yungas'). Tropical & subtropical moist broadleaf forests. Eastern slope of the Andes. Steep slopes, ridges and valleys. Moderately temperate climate at high elevations. Ecoregion ID = 493.	
	Eastern Cordillera Real montane forests (‘andes_eastCR’). Eastern slope of central Andes. Humid tropical climate. Ecoregion ID = 460.	
Rocky Mountains	Colorado Rockies forests ('rockies_colorado'). Highest mountains in the Rocky Mountains. Dry continental and alpine climate. Ecoregion ID = 353.	US Fish and Wildlife Service^27^ (2024)
	South Central Rockies forests (‘rockies_wyoming’). Dry continental climate. Short summers and long cold winters. Ecoregion ID = 367.	
Alps	Alps, conifer and mixed forests (‘alps_west’, ‘alps_center’, ‘alps_east’). Temperate with marked snow/ice accumulation and melting season. Ecoregion ID = 689.	Swiss Federal Office of the Environment^28^ (2011)
High Mountain Asia	Eastern Himalaya alpine shrub and meadows (‘east_himalaya’). Highly seasonal climate with monsoon rainfall from May-September. Rain shadow blocks precipitation in some regions. Ecoregion ID = 751.	Mapped using VHR satellite imagery by Jan Kropáček (2024)
	Tibetan Plateau alpine shrublands and meadows (‘tibet_plateau’). Ecoregion ID = 768.

#### Andes

To delineate training regions for wetland and non-wetland areas in the five Andean test sites (Fig. 2a), we used the publicly available wetland data from the National Bofedales Inventory (2023, 2^nd^ version) (https://www.arcgis.com/home/item.html?id=ebaf41045a1c4a85920aae74b46e0ff2), published by the National Institute for Research in Glaciers and Mountain Ecosystems (INAIGEM)^8,26^. The data set includes high mountain wetland (‘bofedales’) extent across the Peruvian Andes. It was created following a remote sensing-based classification approach, accounting for wetland characteristics in diverse Andean ecoregions. The INAIGEM map was validated using 1537 ground-truth points distributed across the three major bofedales regions and characteristic types in Peru, which were sampled during an extensive *in-situ* data campaign. Validation results of the INAIGEM-mapping approach show an overall accuracy of 85% (Kappa index = 0.71)^8^. To account for the high variety of wetland ecosystems in the Andes, we chose test sites from the Andean Páramo ecoregion, the Peruvian Yungas, the Eastern Cordillera Real Mountain Forest, the Central Andean wet Puna and the Central Andean Puna (Table 1). We sampled our training data from regions above the tree line, at altitudes above 3500 m asl.

#### Rocky Mountains

Two test sites were chosen in the Rocky Mountains, covering the alpine ecoregions of the Colorado Rockies forests and the South-Central Rockies forests (Fig. 2b). Data for wetland extents in these regions was taken from the National Wetland Inventory of the U.S. Fish and Wildlife Service^27^. From the data sets of ‘seamless wetlands data’ (https://www.fws.gov/program/national-wetlands-inventory/download-state-wetlands-data), we selected the Colorado and the Wyoming geodatabases and extracted wetland information for ‘freshwater emergent wetlands’ and ‘freshwater forested/shrub wetlands’. We sampled training and validation points from regions above the tree line at 2000 m asl. We excluded open water bodies and rivers from this selection.

#### Alps

The Swiss REN-data set of wetlands, ‘Nationales ökologisches Netzwerk REN, Lebensraum Feuchtgebiet’^28^ served as training data for wetland regions in the Alps. The data set is published by the Swiss Federal Office of the Environment (BAFU) and available through the Swiss federal geoportal (https://data.geo.admin.ch/browser/index.html#/collections/ch.bafu.ren-extensive_landwirtschaftsgebiete/items/ren-extensive_landwirtschaftsgebiete?.language=en&.asset=asset-ren-extensive_landwirtschaftsgebiete_2056-shp-zip). From the REN wetland data set we selected the categories of ‘central wetland’ and ‘wetland’ (labelled in the data set as ‘Kerngebiete’ and ‘Humide’) to define the wetland extend in our Alp test sites. The Alps cover only one ecoregion, termed as ‘Conifer and Mixed Forest’^25^. As wetland extents in the Alps are small compared to the remaining test sites, we chose three test sites from this ecoregion (Fig. 2c). Sampling from only one small region would substantially limit our sample set to locally specific wetland characteristics. By choosing three regions from the western, central and eastern Swiss Alps we attempt to cover a broader range of Alpine wetland features, to increase the precision of the final classification results. In the Alps we classified high mountain wetlands above a tree line threshold of 2000 m asl.

#### High Mountain Asia

For High Mountain Asia (Fig. 2d), no existing wetland map of sufficient spatial resolution for training could be found. We therefore manually delineated wetland occurrences in two regions, where co-authors of this study conducted research in the past and gained knowledge of local wetland occurrences during three field campaigns in the period 2008–2011. The training polygons were delineated based on vegetation presence in dry period, position in the terrain and a typical spatial pattern involving hummocks, ponds and meandering streams. High-Resolution satellite imagery (VHR satellite) by Maxar available in Google Earth was used for the mapping. We sampled training and validation points above the tree line of 3500 m asl and from the ecoregions of the ‘Eastern Himalaya alpine shrublands and meadows’ and the ‘Tibetan Plateau alpine shrublands and meadows’.

For each of our 12 test sites, we conducted a stratified random sample point selection of 1,500 sample points with wetlands and 1,500 points without wetlands. Points with missing data for any of the predictors were removed, which results in a final data set of 35,515 data points. This data set was split to use 80% of the data to train the classifier and the remaining 20% of the data for validation.

### Input data and predictor variables

The predictors for the classification model were derived from ESA’s Sentinel-1 SAR GRD (orthorectified and calibrated C-band synthetic aperture radar)^18,19^ and the Harmonized Sentinel-2 MSI Level-2A (orthorectified optical imagery at surface reflectance)^20,21^ satellite imagery, from NASA SRTM 30 m digital elevation model (v3)^22,23^, and the RESOLVE data set presenting a global map of ecoregions^25^. All data sets are publicly available in the Google Earth Engine Data Catalogue.

The following image processing was done prior to using the scenes to derive the required spectral information for the predictors: To use the Sentinel-1 SAR GRD images in our classification approach we: i. masked the images to the four selected mountain regions; ii. filtered the scenes to use only those taken with incident angles between 30 and 45 degrees; iii. converted the unit of the backscatter signal from sigma naught to gamma naught, to reduce terrain-induced distortions; and iv. conducted a sigma-lee-filtering to remove the speckle noise, typical for high resolution radar images. The Sentinel-2 scenes were: i. masked to the four mountain regions; ii. filtered to allow only the processing of images with cloud cover < 10%; and iii. averaged over all acquired scenes. This reduced noise and enabled the extraction of a stable spectral signal. In addition, we filtered all images (Sentinel-1 and Sentinel-2) for the Rocky Mountains, the Alps and High Mountain Asia by date and selected only those images taken between June 1^st^ – November 1^st^, for the years 2019–2024. In this way we avoided snow cover which might lead to misclassification. For the Andes we selected all scenes from January 1^st^ – December 31^st^, for the years 2019–2024.

After the pre-processing of the input data files, we derived 12 predictor variables from the acquired scenes. Their respective relevance for capturing high altitude wetland features is listed in Table 2. The selection is based on predictors which have been proven suitable for wetland detections in previous studies^16^. To account for the high heterogeneity of mountain wetlands across the selected four mountain regions, we chose a combination of predictors derived from radar backscatter (Sentinel-1) and optical and multispectral (Sentinel-2) imagery, topographic and ecological variables. While indices based on optical and multispectral sensor data can best capture vegetation dynamics, radar backscatter and topographic indices better reveal spatially distributed moisture dynamics^29^. Including ecoregions as a separate indicator allows the model to distinguish between different wetland characteristics per ecoregion and indirectly includes regionally specific climate and vegetation characteristics.

**Table 2:** Predictor variables and their relevance for wetland mapping.

Predictor variable	Description and relevance for identifying high mountain wetlands	Equation or value (b = Sentinel-2 band)	Data source
VV (γ^0^)	Vertical polarization of backscatter signal converted from sigma-0 (σ^0^) to gamma-0 (γ^0^) coefficient to account for terrain-induced distortions. VV is sensitive to surface moisture content and open water^39^.	${{VV}}_{{\rm{g}}0}=\frac{{{VV}}_{{\rm{s}}0}}{\cos ({incidence\; angle})}$	Sentinel-1
VH (γ^0^)	Cross polarization of backscatter signal converted to gamma-0 coefficient. VH is sensitive to surface structures, e.g. vegetation structures over wet soils^39^.	${{VH}}_{{\rm{g}}0}=\frac{{{VH}}_{{\rm{s}}0}}{\cos ({incidence\; angle})}$	Sentinel-1
NDPI	Normalized difference polarization index. Combining information from VV and VH to better distinguish moisture and vegetation characteristics over wetlands^16^.	$\left(\frac{{VH}-{VV}}{{VH}+{VV}}\right)$	Sentinel-1
ARI	Anthocyanin reflectance index. This index is sensitive to anthocyanin pigments in leaves which is often linked to plant water stress^16^.	$\left(\frac{b8}{b2}\right)-\left(\frac{b8}{b3}\right)$	Sentinel-2
NDVI	Normalized difference vegetation index. Indicates photosynthetic activity. Efficiently distinguishes vegetated areas from sparse non-vegetated areas^16^.	$\left(\frac{b8-b4}{b8+b4}\right)$	Sentinel-2
NDWI	Normalized difference water index. Indicator sensitive to moisture conditions, such as high surface saturation^16^.	$\left(\frac{b3-b8}{b3+b8}\right)$	Sentinel-2
REIP	Red edge inflection point. An approximation on a hyperspectral index for estimating the position (in nm) of the NIR/red inflection point in vegetation spectra^16^.	$702+40\left(\frac{\left(\frac{b4-b7}{2}\right)-b5}{(b6-b5)}\right)$	Sentinel-2
TCG	Tasselled cap greenness index. Extracts differences of phenological characteristics of vegetation^40,41^. Like NDVI effective in detecting green areas in bare high mountain regions.	$-(0.2941\times b2)-(0.243\times b3)-(0.5424\times b4)+(0.7276\times b8)+(0.0713\times b11)-(0.1608\times b12)$	Sentinel-2
Ecoregion	Ecoregion indicating locally specific climate and vegetation characteristics. We use the Eco_ID of the RESOLVE data set^25^ to differentiate between ecoregions.	Eco_ID	RESOLVE
Elevation	Elevation [m] above sea level. Indication of a ‘high’ mountain wetland if > regional tree line.	>regional tree line	SRTM-DEM
Slope	Slope angle in degrees. Indicator for steepness of topography. The higher the slope the less likely the formation of wetlands.	—	SRTM-DEM
TPI	Topographic positioning index. Indicates the topographic position of a pixel relative to the elevation of its surrounding pixels (${Z}_{(i+k,j+l)}$). We use a pixel surrounding of 5 × 5 pixels and a DEM of 30 m resolution.	${TPI}={elev}.-\frac{1}{25}\sum {Z}_{(i+k,j+l)}$	SRTM-DEM

### Classification algorithm

The classifier is a random forest machine learning model using the selected data set of our 12 predictors and trained on sample points at 28,412 locations (i.e., 80% of 35,515 sample points). We use Google Earth Engine’s inbuilt ‘smileRandomForest’ function^30^, to implement the model. Hyperparameters for the model construction are set according to commonly suggested model setups^31^ and according to the trade-off between model performance and computation costs: Number of trees: 50; variables per split: 3 (square root of our 12 predictors rounded down); minimum leaf population: 1. To maintain an operational model which is not bounded by computational constraints, we deliberately kept the numbers of maximum allowed trees at 50. This allowed us to minimize the number of model runs, while generating a large-enough ensemble to produce stable predictions and reliable probabilities of wetland detection. Running the model with a higher tree-thresholds of up to 100 trees did not yield a significant improvement of the model accuracy but in substantially higher computational demand. The selection of three predictor variables per split, keeps each tree diverse, decorrelates trees and reduces overfitting. Reducing the number of variables per split might increase bias, and increasing the number might increase correlation between trees. The rationale behind the minimum leaf population of 1, is to allow the trees to grow deep. As we run a global classification over heterogenous regions, this allows us to capture local variability in our wetland classes. To ensure the accuracy and robustness of the classifier despite our rather low tree count, we run a k-fold cross validation (see section on technical validation).

## Data Records

The maps and the code for the classification procedure are stored in a data repository on Zenodo and can freely be downloaded from: https://zenodo.org/records/18339573.41. The data is named and formatted as shown in Table 3.

**Table 3:** List of data records (High_mountain_wetlands_v1.1.zip).

Data Records	
**Maps_full_probabilities**	
High_Mountain_Wetlands_Alps_full_prob.tif	The data contains the classified wetland map for each of the four selected mountain regions. It shows the average wetland extent for the years 2019–2024. Pixel values are 0-1 (‘full_prob’) and 0.7–1.0 (‘high_prob’), with 0 indicating ‘no-wetland’, and >0 indicating pixel-wise wetland probability.
High_Mountain_Wetlands_Andes_full_prob.tif	
High_Mountain_Wetlands_HighMountainAsia_full_prob.tif	
High_Mountain_Wetlands_RockyMountains_full_prob.tif	
**Maps_high_probabilities**	
High_Mountain_Wetlands_Andes_high_prob.tif	The data contains the classified wetland map for each of the four selected mountain regions. It shows the average wetland extent for the years 2019–2024. Pixel values are 0-1 (‘full_prob’) and 0.7–1.0 (‘high_prob’), with 0 indicating ‘no-wetland’, and >0 indicating pixel-wise wetland probability
High_Mountain_Wetlands_Alps_high_prob.tif	
High_Mountain_Wetlands_HighMountainAsia_high_prob.tif	
High_Mountain_Wetlands_RockyMountains_high_prob.tif	
**Data**	
Training_area_extents	Folder containing shapefiles of rectangular extents of each training region.
Training_areas_non_wetlands	Folder containing shapefiles of non-wetland sites, used for sampling training points in areas without wetlands.
Training_areas_wetlands	Folder contining shapefiles of high mountain wetlands, used to sample training points in areas with wetlands.
**Metric**	
area_adjusted_by_region.csv	Tables with results from global and region-specific accuacary assements, including overall accuracy, area adjusted accuracy, leave-one-site-out (LOSO) and leave-one-region-out (LORO) cross validation.
loso_cv_metrics.csv	
loro_cv_metrics.csv	
**Model**	
Step_1_Sample_training_data.ipynb	Code to load and preprocess the training data, to derive the wetland indicators and to train the classifier
Step_2A_Kfold_cross_validation.ipynb Step_2B_LOSO_LORO_cross_validation.ipynb	Code to perform the cross validation and accuracy assessments for global and regional data sets.
Step_3_Applying_the_classifier.ipynb	Code to apply the classifier to classify high mountain wetlands.
**Code**	
Merge_tiles_and_calculate_area.py	Code to join single tiles, reproject maps and calculate total areal extent of wetlands.
**Mapping Conventions**	
readme.txt	Readme file with mapping conventions

## Technical Validation

The validation of the wetland classification was done in a two-step procedure.

1. Testing the accuracy and the robustness of the classification.
2. Assessing the robustness of the probability maps.

### Step 1: Testing the accuracy and robustness of the classification

The accuracy and robustness of the classifier was tested in a k-fold cross validation procedure^32^. Using five folds, the classifier was trained and subsequently validated five times, each time with a new and independently selected subset of training and validation data. We sampled the validation data from the same test sites used for training (Fig. 2, white boxes), yet, at different geographic locations to ensure independence between training and validation data. Test sites cover on average an area of 2870 km^2^ and spread over regions with various topographical, botanical and hydrological surface characteristics (Table 1, Table S.1). Validation data therefore accounts for heterogeneous wetland sites and allows for assessing the classification accuracy of wetlands across all considered ecoregions. We tested for the prediction accuracy of each fold and for a consistent accuracy over all folds, to judge the robustness of the classifier and to rule out potential overfitting and biases towards the respective training data selection. We calculated the accuracy metrics globally as well as regionally to identify geographical variations in the classification performance.

The global cross-validation results show consistent overall accuracies of 85%-86% for the classification of the global map (Table 4). These results are comparable to benchmark accuracies from global land cover mapping studies^33^, and reach the commonly set threshold considered as ‘good’ classification accuracy of >85%^34^. The consistency of the accuracies across all folds indicates that the classification error is largely independent of the given training data set, the model predictions are persistent across folds, and model overfitting can be ruled out. This also indicates that our training data set is sufficiently heterogeneous to construct a classifier which generalises well across different data subsets. This is an important requirement for the application of the classifier for our wetland classifications across different ecoregions.

**Table 4:** Mean, minimum and maximum classification accuracies across all 5 folds and for all regions.

Overall classification accuracies			
	Mean [%]	Min [%]	Max [%]
Global (all four mountain regions)	**86**	85	86
Andes	**91**	90	91
Rocky Mountains	**83**	82	84
Alps	**76**	75	78
High Mountain Asia	**93**	92	93

Consistent accuracies for wetland classifications in each mountain region, prove the robustness of our classifier also for regional classifications. Regional accuracies however vary. The highest accuracies can be achieved for the Andes (mean: 91%; range: 90%-91%) and for High Mountain Asia (mean: 93%; range: 92%-93%). Classification results for the Rocky Mountains show average accuracies of 83% (range: 82%-84%). Results for the Alps show lowest accuracies of 76% (range: 75%-78%). Confusion matrices specifying the user’s accuracy (commission error), producer’s accuracy (omission error), and the overall accuracy^35^ of our wetland classifications, globally as well as regionally, can be found in the Supplementary Information (see Fig. S.1. – Fig. S.5.).

In addition to the k-fold cross validation, we perform a leave-one-site-out (LOSO) and a more stringent leave-one-region-out (LORO) cross-validation. Both approaches explicitly enforce spatial independence between training and test sets and account for the fact, that samples within the same training site and region might originate from highly similar environmental conditions, and might introduce spatial bias into the training data set. In Tables 5 and 6, we report per-class user’s and producer’s accuracies (UA and PA), as well as the F1 score and Intersection over Union (IoU; see SI for detailed equations). We also report the balanced accuracy to provide a class-weight-independent measure of performance. This metric ensures that both classes contribute equally to the overall accuracy estimate, regardless of their relative classification difficulty.

**Table 5:** Leave-one-site-out (LOSO) cross-validation results.

Training Site*	UA_0	UA_1	PA_0	PA_1	F1_0	F1_1	IoU_0	IoU_1	Balanced accuracy	Overall accuracy
alps_center	0.58	0.67	0.80	0.41	0.67	0.51	0.50	0.34	0.60	0.60
alps_east	0.64	0.65	0.67	0.62	0.65	0.64	0.49	0.47	0.65	0.65
alps_west	0.55	0.73	0.90	0.27	0.68	0.39	0.52	0.24	0.58	0.58
andes_eastCR	0.53	0.54	0.82	0.22	0.65	0.31	0.48	0.19	0.52	0.53
andes_paramo	0.95	0.84	0.82	0.96	0.88	0.90	0.79	0.81	0.89	0.89
andes_yungas	0.95	0.88	0.87	0.95	0.91	0.92	0.83	0.84	0.91	0.91
andes_wet_puna	0.88	0.84	0.83	0.89	0.86	0.87	0.75	0.76	0.86	0.86
andes_puna	0.69	0.96	0.97	0.57	0.81	0.71	0.68	0.55	0.77	0.77
rockies_colorado	0.71	0.80	0.83	0.66	0.77	0.72	0.62	0.57	0.75	0.75
rockies_wyoming	0.77	0.83	0.85	0.75	0.81	0.79	0.68	0.65	0.80	0.80
east_himalaya	0.67	0.90	0.94	0.54	0.78	0.67	0.64	0.51	0.74	0.74
tibet_plateau	0.67	0.95	0.97	0.53	0.79	0.68	0.66	0.51	0.75	0.75

^*^see Fig. 1 for training site locations and Table 1 for details on the respective climate and ecosystem characteristics. Prefix 0 (e.g. UA_0) = non-wetland; Prefix 1 (UA_1) = wetland class.

**Table 6:** Leave-one-region-out (LORO) cross-validation results.

Region	UA_0	UA_1	PA_0	PA_1	F1_0	F1_1	IoU_0	IoU_1	Balanced accuracy	Overall accuracy
Andes	0.73	0.85	0.89	0.67	0.80	0.75	0.67	0.60	0.78	0.78
Rocky M.*	0.58	0.73	0.86	0.39	0.70	0.51	0.53	0.34	0.62	0.62
Alps	0.52	0.72	0.95	0.13	0.67	0.23	0.51	0.13	0.54	0.54
HMA**	0.66	0.93	0.96	0.52	0.79	0.66	0.65	0.50	0.74	0.74

^*^Rocky Mountains; **High Mountain Asia; Prefix 0 (e.g. UA_0) = non-wetland; Prefix 1 (UA_1) = wetland class.

Finally, we calculated the region-specific, area-adjusted overall accuracy, which corrects for the class imbalance and prevents overall accuracy from being dominated by the more extensive non-wetland class. This provides a fairer regional evaluation than the simple Overall Accuracy (Table 4).

LOSO cross validation (Table 5) shows strong spatial transferability across most Andean, Rockies and Himalaya test sites (Balanced Accuracy 0.74–0.91, F1₁ 0.67–0.92). However, generalization to Alpine sites is notably weaker, particularly due to low producer’s accuracy for our wetland class (PA₁ = 0.27–0.62), indicating substantial omission of wetlands when trained on other regions. Leave-one-region-out (LORO) cross-validation (Table 6) revealed substantial differences in spatial transferability across mountain regions. When training on all other regions, predictions in the Andes and HMA remain comparatively strong (Balanced Accuracy = 0.78 and 0.74, respectively), with high precision for wetlands (UA₁ = 0.85–0.93). In contrast, performance in the Rocky Mountains and, most notably, the Alps is markedly reduced (Balanced Accuracy = 0.62 and 0.54), primarily due to low producer’s accuracy for the wetland class (PA₁ = 0.39 and 0.13), indicating substantial omission of wetlands in these regions. Overall, the results suggest that model generalization across continents is feasible for the Andes and HMA, but transferability is limited for the Alps and partly for the Rockies. Across regions, the area-adjusted accuracy (Table 7) confirms the findings in Tables 4 and 6. Highest accuracies can be achieved for the Andes (0.74) and HMA (0.69), intermediate accuracies for the Rocky Mountains (0.59) and lowest accuracies for the Alps (0.53). This means that even after adjusting for regional class proportions and map area, generalization performance varies substantially among mountain regions. Transferability is clearly best in the Andes and HMA and weakest in the Alps, consistent with the cross-validation metrics.

**Table 7:** Region-specific area-adjusted overall accucary.

Region	Area adjusted overall accuracy	95% - confidence interval
Andes	0.74	0.011
Rocky Mountains	0.59	0.009
Alps	0.53	0.014
High Mountain Asia	0.69	0.013

### Step 2: Assessing the robustness of the probability maps

**Fig. 3:**
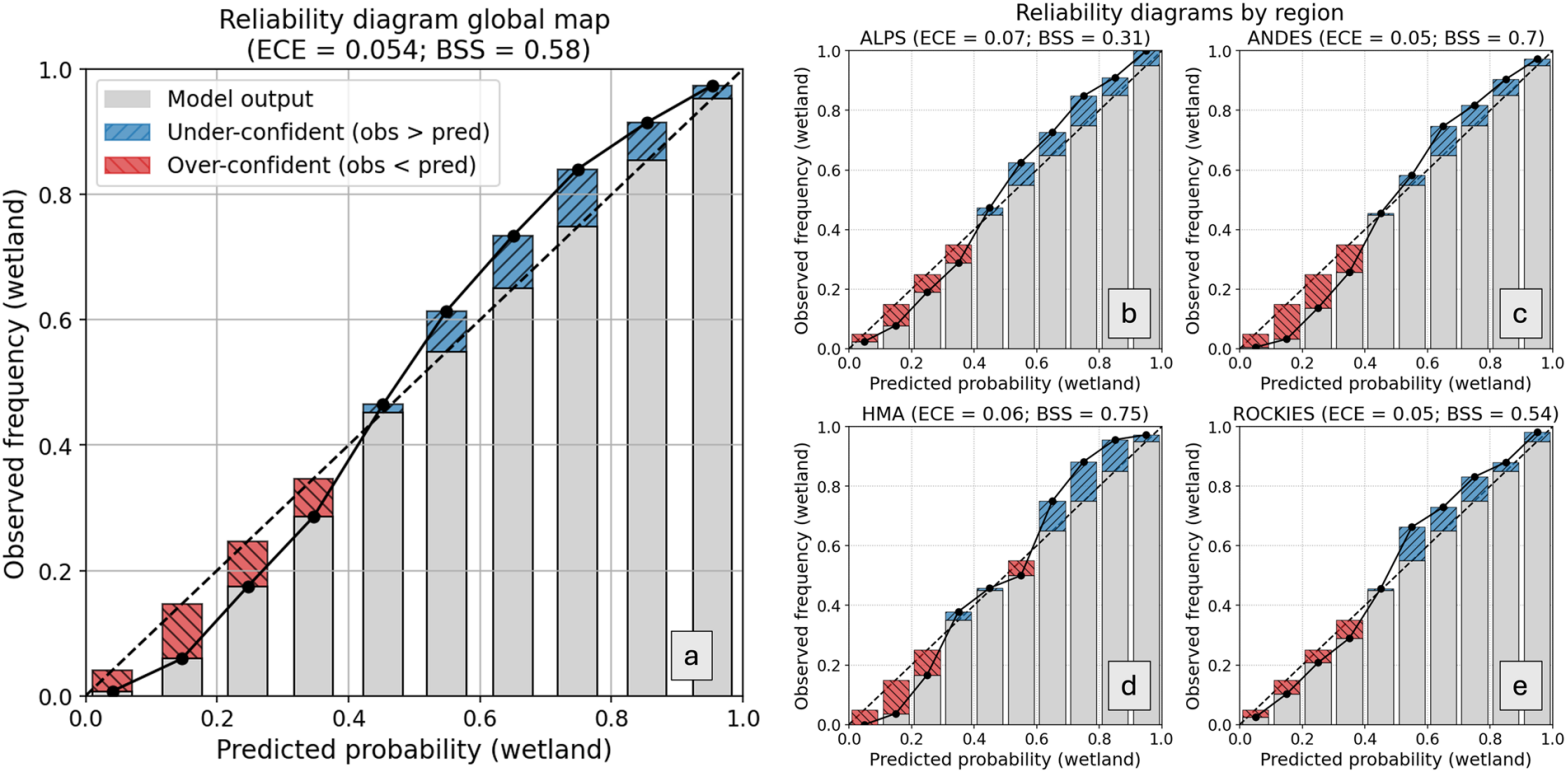
Reliability diagrams for global as well as regional-specific model performances.

To assess how well our predicted wetland probabilities reflect empirical frequencies, we examined the reliability of our probabilistic outputs at global and regional scales. Reliability diagrams (Fig. 3a–e), show that predicted wetland probabilities are generally well calibrated at the global scale (Expected Calibration Error (ECE) ≈ 0.05; Brier Skill Score (BSS) ≈ 0.58), with predicted probabilities closely matching observed frequencies. Regional-specific results show excellent calibration in the Andes (Fig. 3c) and High Mountain Asia (Fig. 3d) (ECE ≤ 0.06, BSS ≥ 0.70), moderate calibration in the Rockies (Fig. 3e), and noticeably poorer calibration in the Alps (Fig. 3b) (ECE = 0.07; BSS = 0.31). This is consistent with the lower model transferability for the Alps, shown in the previous validation results.

To assess the robustness (or “certainty”) of the estimated total wetland area in each mountain region, we focused on wetlands classified with probabilities of 70% or higher (Table 8).

**Table 8:** Total area of classified wetlands for three certainty classes.

Wetland Area [km^2^]	Certainty of wetland detection			
	Low certainty (70% − < 80%)	Medium certainty (80% − < 90%)	High certainty (90%–100%)	Total (all classes)
Andes	11,996	9,014	7,578	28,590
Rocky Mountains	19,925	14,465	6,190	40,580
Alps	1,232	283	15	1,431
High Mountain Asia	23,689	23,631	16,777	64,099
***Total wetland area:***	***56,842 km***^***2***^	***47,393 km***^***2***^	***30,560 km***^***2***^	***134,700 km***^***2***^

**Fig. 4:**
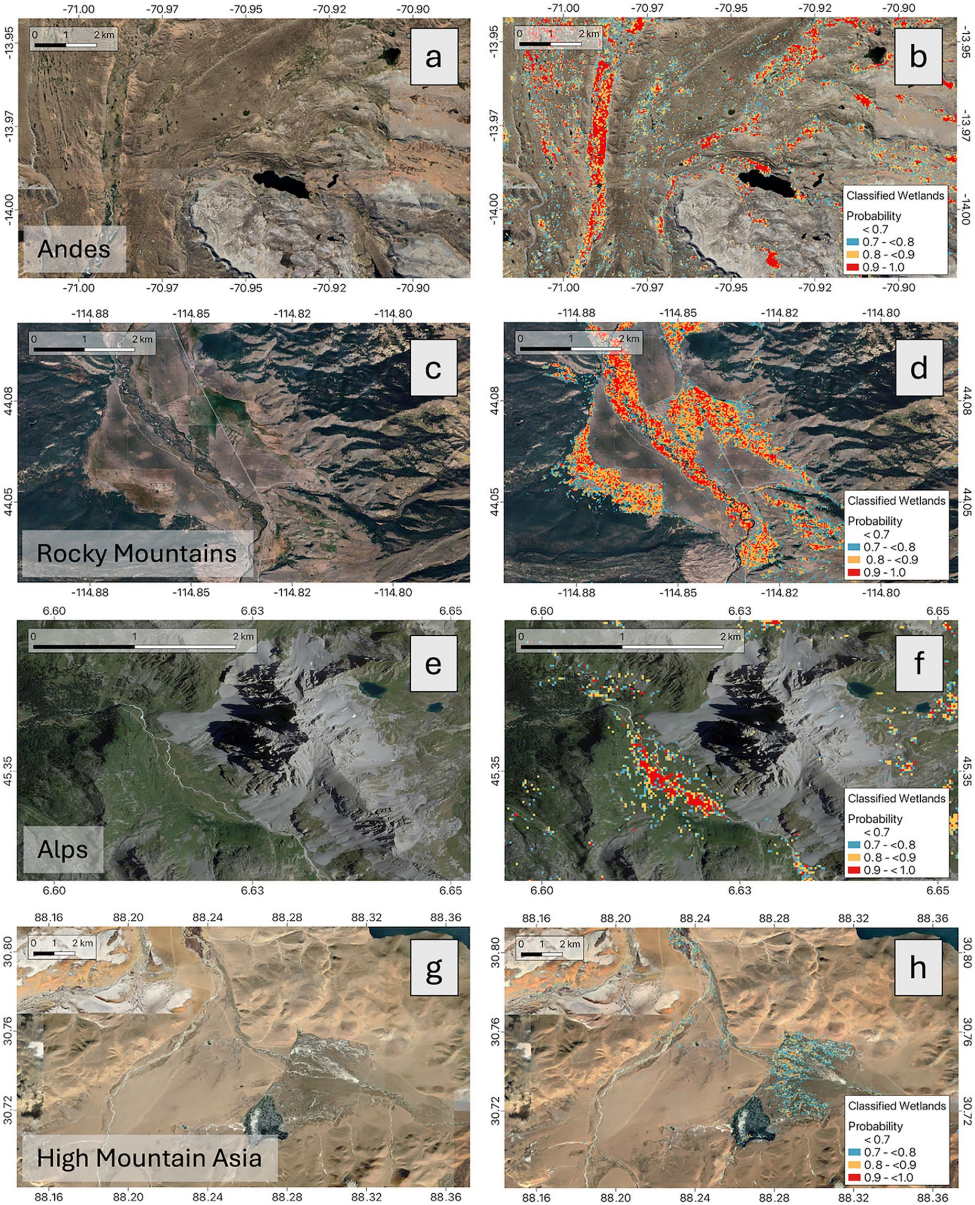
Left column: images of selected mountain regions without classification results. Right column: images with classification results, showing the wetland map with classification probabilities of >70%.

The results align with the accuracy assessments. In the Andes and HMA, 27% and 26% of wetlands, respectively, are classified with probabilities of 90% or higher (Table 5). In the Rocky Mountains, approximately 16% of wetlands fall into this high-certainty category. The Alps exhibit the lowest proportion of “highly likely wetlands”, with only 1% classified with probabilities above 90%. These differences are likely influenced by factors such as the unique characteristics of ecoregions (i.e. distinctive features between wetlands and non-wetlands, fragmentation of wetlands, seasonality of hydrological dynamics), the varying sizes of wetlands, and most importantly the availability and distribution of training data. The factors collectively affect the clarity and certainty of wetland classification in different mountain regions. E.g. the low certainty of wetland classification in the Alps can likely be ascribed to the fact that wetlands are more fragmented and significantly smaller than e.g. in the broader inter-Andean valleys and on the Tibetan Plateau, which complicates their classification^36^. Their vegetation also often resembles surrounding meadows, hindering the detection of clear spectral wetlands-signatures (Fig. 4e-f). Steep slopes and high terrain variability in the Alps might create shadow effects and disturbances in the reflectance/backscatter values of satellite data^36^. Furthermore, alpine wetlands might receive a higher moisture contribution from precipitation and snow and glacier melt^37^ compared to ground water fed wetlands (e.g. in the Andes). This leads to high variability and intermittent surface saturation, making a clear detection of wetlands in this environment particularly challenging.

The total area of wetlands classified with a probability of 90% and higher, sums up to >30,500 km^2^ (Table 8). This represents a significant carbon reservoir and a critical hydrological feature in regions increasingly affected by diminishing water supplies due to glacier and snow melt reduction. Hence, our map underlines the extent and importance of these unique high mountain ecosystems, which are often overlooked due to their remote and hidden locations. It presents a first step towards improving our knowledge of high mountain water resources and high mountain wetland dynamics. It allows to advance research which aims to explore the role of high mountain wetlands in securing a safe water supply for mountain communities, their global importance as carbon stores, and their value as biodiversity hotspots.

## Usage Notes

The map has been created using all available satellite imagery between January 1, 2019 and December 6, 2024 (last day of data retrieval), following the data selection criteria outlined above (e.g., cloud cover, specific selection of dates, and specific incident angles). However, despite the length of this training period, image coverage varies across regions. Areas with lower image coverage are more prone to artifacts, which is most pronounced on the borders between neigbouring images that stem from significantly different dates. While these image inconsistencies cannot be resolved at this stage, they will reduce with time as more images become available.

We note a recurring source of commission error in regions, where irrigated agricultural fields can resemble wetlands in spectral and structural characteristics. Because our approach is intentionally global and region-agnostic, we do not introduce region-specific training classes (e.g., irrigated agriculture), which would improve results locally but reduce the method’s generalizability. Agricultural areas are limited in extent within our high-mountain study domain, but users should be aware that misclassifications may occur in regions where agriculture and wetlands co-occur.

Due to computational constraints, our mapping approach did not allow for radiometric terrain and topographic illumination correction. In steep terrain, this can introduce topography-driven biases due to different spectral signals which might degrade classification accuracy and reliability. This should be considered particularly in areas with high elevation gradients, steep slopes and narrow valleys.

Training areas for High Mountain Asia are relatively small compared to the size of the entire mountain region and do not fully represent its diverse ecoregions. Although classification accuracy at the training sites reaches 92%, visual inspection reveals a substantial overestimation of wetlands in the eastern part of the region, particularly in the Southeast Tibetean Shrublands. Expanding the training data set to include areas with very distinct climatic and hydrological characteristics can reduce this issue further.

## Supplementary information

Supplementary Information

## Data Availability

The maps and the code for the classification procedure are stored in a data repository on Zenodo and can freely be downloaded from: https://zenodo.org/records/18339573^38^. The repository contains seven sub-folders (as listed in Table 3): The folders ‘Maps_full_probabilities.zip’ and ‘Maps_high_probabilities.zip’ contain the classified wetland maps for each of the four selected mountain regions (Alps, Andes, Rocky Mountains and High Mountain Asia) showing the full probabilities (i.e. 0–100%) and high probabilities (70%–100%), with which each pixel is classified as a wetland pixel. The ‘Data.zip’ folder contains the shapefiles of the study regions boundaries and the shapefiles for the regions used to sample training and validation data points. In the ‘Metric.zip’ folder we list csv-files containing the results of global and regional-specific accuracy assessments. The ‘Model.zip’ folder contains the code to run the wetland classification model, including all pre-processing, validation and classifications steps. The code used to merge the output and calculate the total wetland extent can be found in the ‘Code.zip’ folder. And finally, mapping conventions can be found in the ‘readme.txt’ file.

## Code availability

The code used to generate and validate the wetland maps is made publicly available under: https://zenodo.org/records/18339573.
