# Supplementary material for: A map of high-altitude wetlands in the world’s major mountain regions
Source: Sci Data. 2026 Mar 11;13:656. doi: 10.1038/s41597-026-07020-w (PMC13111728; doi:10.1038/s41597-026-07020-w)
Supplement: Supplementary file 1 — Supplementary Information [file 41597_2026_7020_MOESM1_ESM.docx]

**Supplementary Information for:**

**A map of high mountain wetlands in the world’s major mountain regions**

Rike Becker^1^, Anthony Ross^1^, Tom Gribbin^2^, Jan Kropáček^3^, Fabian Drenkhan^4,5^, Lilia Hernandez Sotelo^6^, Marc Martinez Mendoza^7^, Bethan Davies^8^, Jeremy Ely^9^, Wouter Buytaert^1^

^1^Department of Civil and Environmental Engineering, Imperial College London, London, UK

^2^British Geological Survey, Environmental Science Centre, Keyworth, UK

^3^Department of Physical Geography and Geoecology, Faculty of Science, Charles University, Prague, Czechia

^4^Geography and Environment, Department of Humanities, Pontificia Universidad Católica del Perú, Lima, Peru

^5^Grupo de Glaciología y Ecohidrología de Montañas Andinas (GEMS), Institute for Nature, Earth and Energy (INTE), Pontificia Universidad Católica del Perú, Lima, Peru

^6^City Planning Labs, The World Bank Group, London, UK

^7^Port de Barcelona, Barcelona, Catalunya, ES.

^8^School of Geography, Politics and Sociology, Newcastle University, Newcastle-upon-Thyne, UK

^9^School of Geography and Planning, The University of Sheffield, Sheffield, UK

**Content of the Supplementary Information**

| Figure and table numbers | Figure and table title | Page number |
| --- | --- | --- |
| Table S.1 | Areal extent of training regions and their coverage by wetlands in the training data sets | 2 |
| Table S.2 | Accuracy metrics | 3 |
| Figures  S.1 – S.5 | Confusion matrices of the k-fold cross validation of each region | 4-6 |

**S.1 – Areal extent of training regions and their coverage by wetlands in the training data sets**

Table S.1: Area of training regions and their respective wetland coverage.

| **Training region** | **Ecoregion ID [RESOVE**^1^ **data set]** | **Area of training region [km^2^]** | **Coverage of wetlands [%]** |
| --- | --- | --- | --- |
| **Andes** |  |  |  |
| Andean Páramo | 590 | 1609 | 5 |
| Central Andean wet Puna | 589 | 1686 | 7 |
| Central Andean Puna | 588 | 1500 | 12 |
| Peruvian Yungas | 493 | 3614 | 1 |
| Eastern Cordillera Real | 460 | 561 | 3 |
| **Rocky Mountains** |  |  |  |
| Colorado Rockies forests | 353 | 13490 | 2 |
| South Central Rockies forests | 376 | 8083 | 4 |
| **Alps** |  |  |  |
| Coniferous forest-1 | 689 | 238 | 3 |
| Coniferous forest-2 | 689 | 126 | 3 |
| Coniferous forest-3 | 689 | 315 | 2 |
| **High Mountain Asia** |  |  |  |
| Eastern Himalaya alpine shrub/meadows | 751 | 1178 | 17 |
| Tibetan Plateau Alpine Shrublands | 768 | 2056 | 3 |

1. Dinerstein, E. *et al.* An Ecoregion-Based Approach to Protecting Half the Terrestrial Realm. *BioScience* **67**, 534–545 (2017).

**S.2 – Accuracy metrics**

Table S.2 – Accuracy metrics (FN=false negative, FP=false positive, TN=true negative, TP=true positive)

| User accuracy (UA) | TP/(TP+FP) |
| --- | --- |
| Producer accuracy (PA) | TP/(TP+FN) |
| Commission error | 1 – UA |
| Omission error | 1 – PA |
| F1 score | (2x (UA x PA))/(UA+PA) |
| IoU (Intersection over Union) | TP/(TP+FP+FN) |
| Overall accuracy (OA) | (TP+TN)/(TP+TN+FP+FN) |
| Balanced accuracy | 0.5((TP/(TP+FN))+(TN/(TN+FP)) |
| Area-weighted overall accuracy | $\sum_{i=1}^{k} w_{i} x {OA}_{i}$ , where ${OA}_{i}=class specific acc.; w_{i}=area proportion$ |

**S.3 - Confusion matrices of the cross validation**

Abbreviations:

Obs. = Observed

User Acc. = User Accuracy (UA)

Prod. Acc. = Producer Accuracy (PA)

Com. Error = Commission Error

Om. Err. = Omission Error


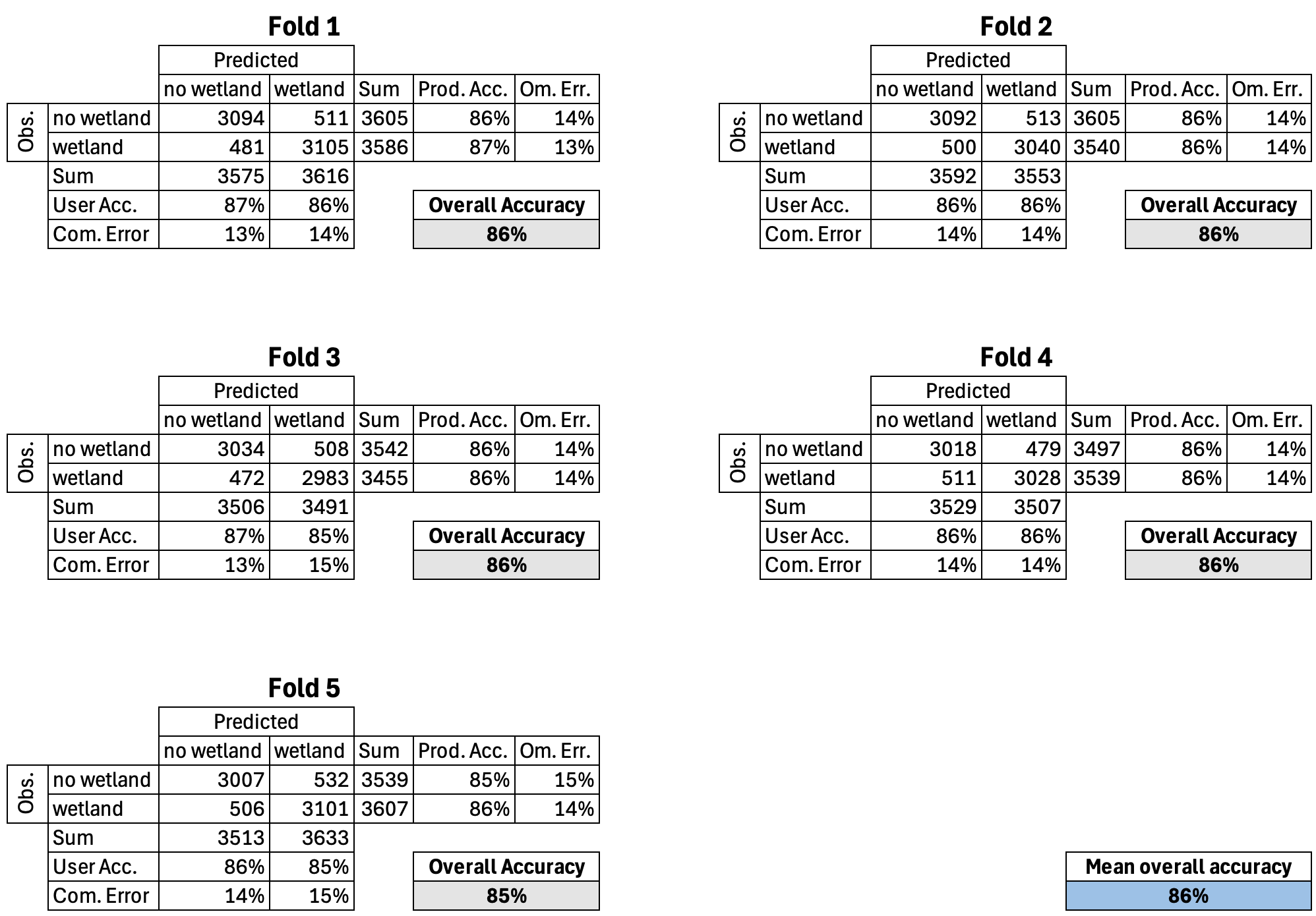


Figure S.1: Confusion matrices of the **global assessment** (classification accuracy across all 4 mountain regions for all 5 folds).


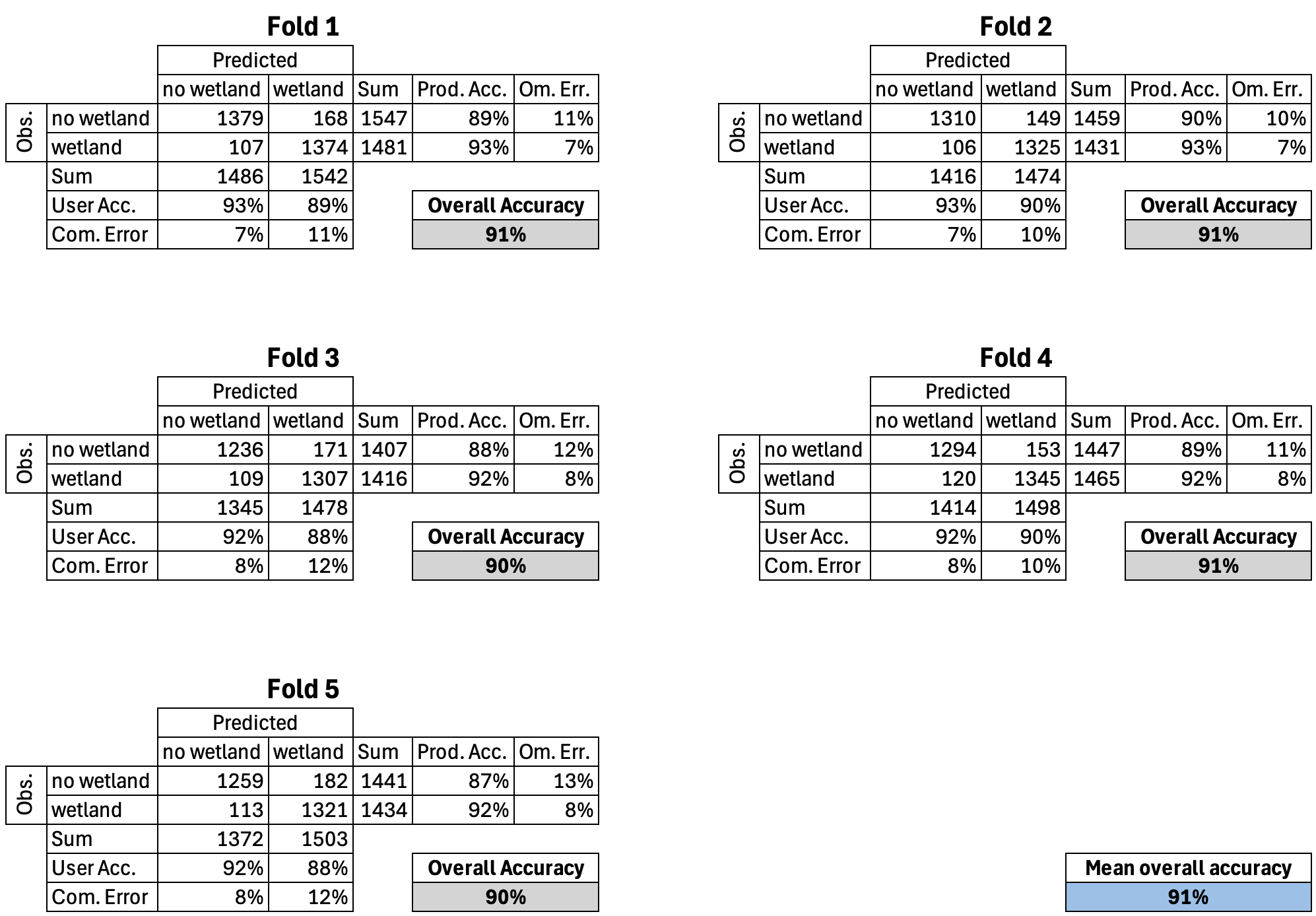


Figure S.2: Confusion matrices for the **Andes** (classification accuracy for all 5 folds).


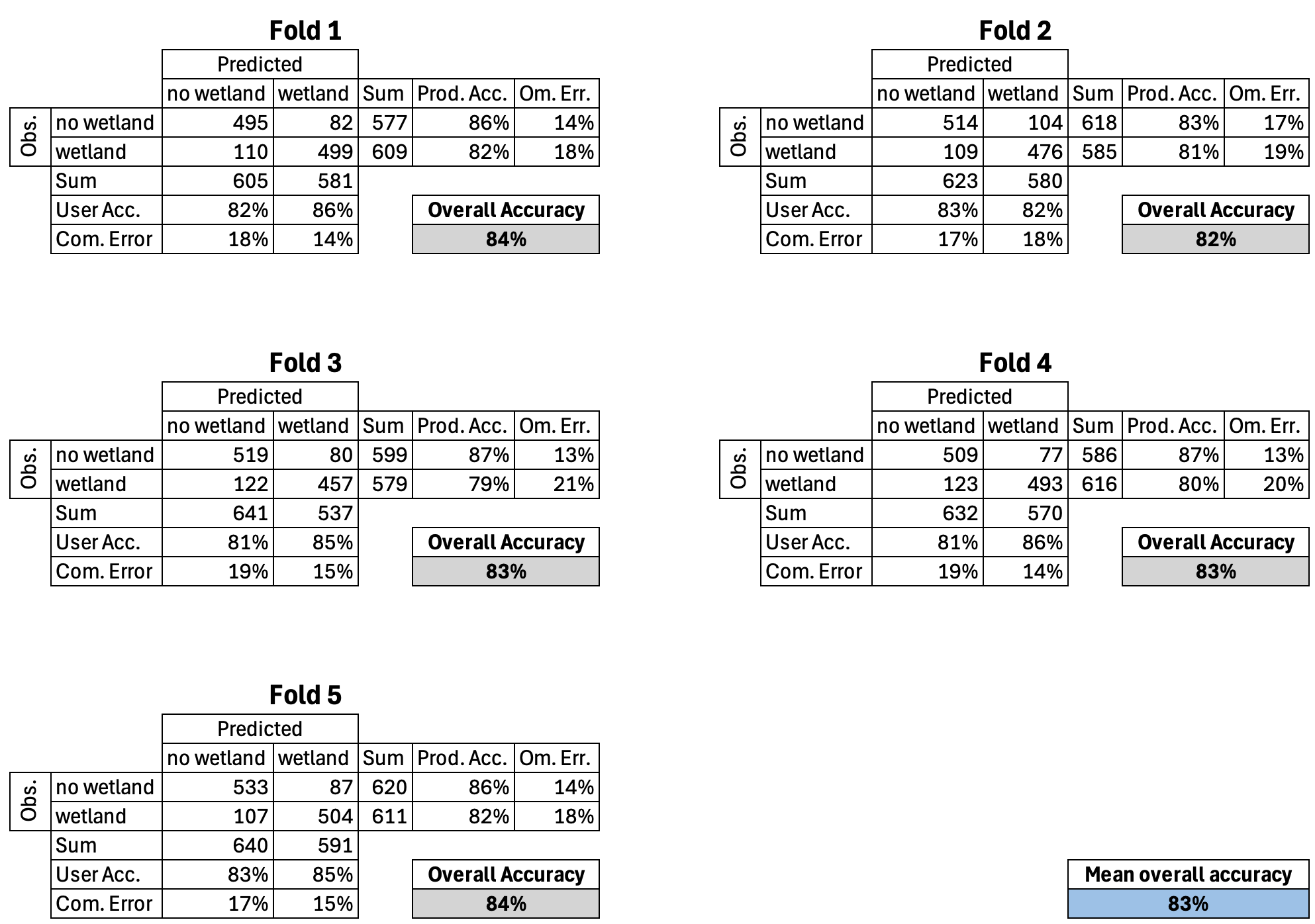


Figure S.3: Confusion matrices for the **Rocky Mountains** (classification accuracy for all 5 folds).


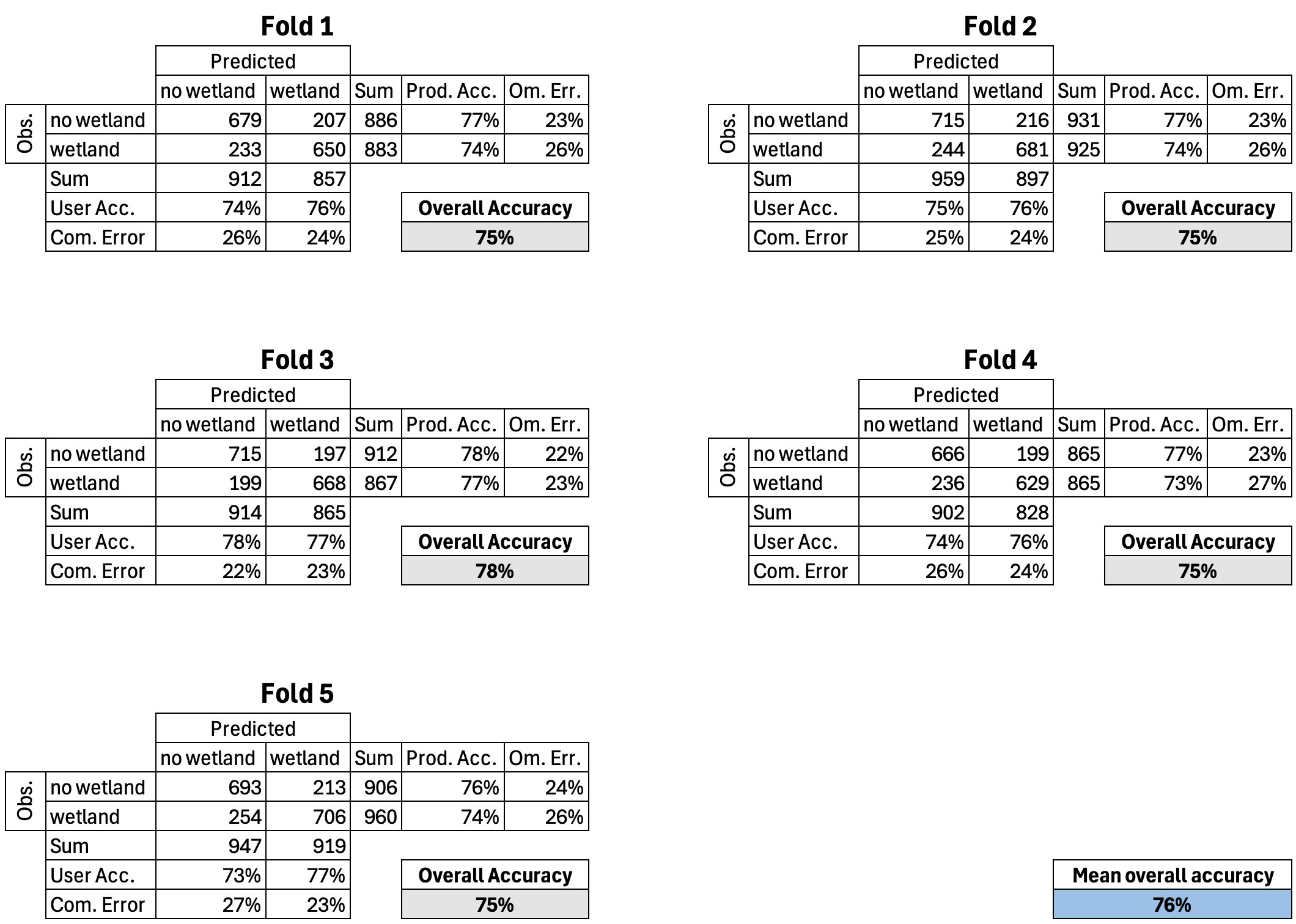


Figure S.4: Confusion matrices for the **Alps** (classification accuracy for all 5 folds).


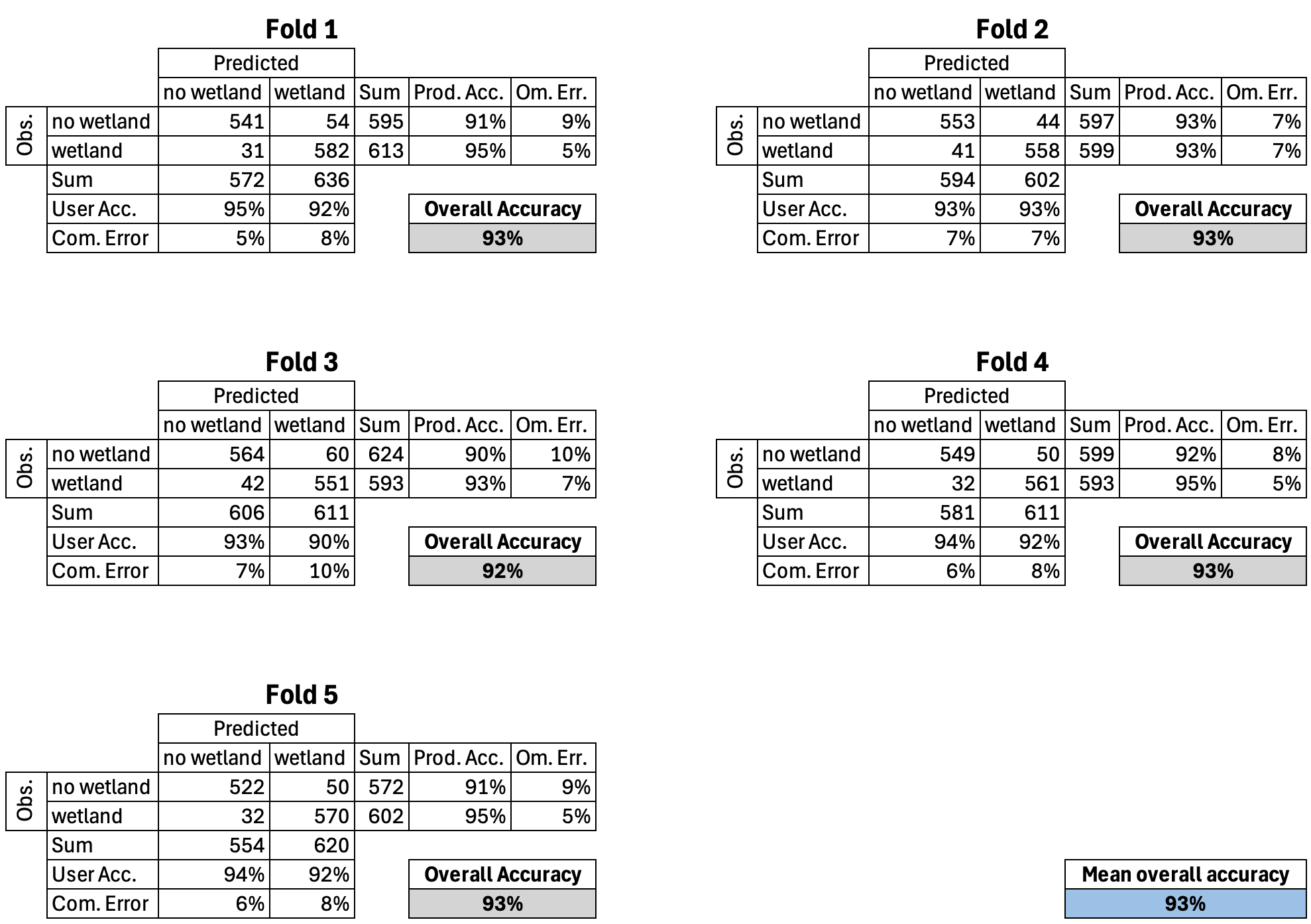


Figure S.5: Confusion matrices for **High Mountain Asia** (classification accuracy for all 5 folds)
